# Individuals’ positive gains from the COVID-19 pandemic: a qualitative study across 30 countries

**DOI:** 10.7189/jogh.15.04091

**Published:** 2025-02-14

**Authors:** Jiaying Li, Patricia M Davidson, Daniel Yee Tak Fong, Yaqin Li, Kris Yuet Wan Lok, Janet Yuen Ha Wong, Mandy Man Ho, Edmond Pui Hang Choi, Vinciya Pandian, Wenjie Duan, Marie Tarrant, Jung Jae Lee, Chia-Chin Lin, Oluwadamilare Akingbade, Khalid M Alabdulwahhab, Mohammad Shakil Ahmad, Mohamed Alboraie, Meshari A Alzahrani, Anil S Bilimale, Sawitree Boonpatcharanon, Samuel Byiringiro, Muhammad Kamil Che Hasan, Luisa Clausi Schettini, Walter Corzo, Josephine M. De Leon, Anjanette S. De Leon, Hiba Deek, Fabio Efficace, Mayssah A El Nayal, Fathiya El-Raey, Eduardo Ensaldo-Carrasco, Pilar Escotorin, Oluwadamilola Agnes Fadodun, Israel Opeyemi Fawole, Yong-Shian Shawn Goh, Devi Irawan, Naimah Ebrahim Khan, Binu Koirala, Ashish Krishna, Cannas Kwok, Tung Thanh Le, Daniela Giambruno Leal, Miguel Ángel Lezana-Fernández, Emery Manirambona, Leandro Cruz Mantoani, Fernando Meneses-González, Iman Elmahdi Mohamed, Madeleine Mukeshimana, Chinh Thi Minh Nguyen, Huong Thi Thanh Nguyen, Khanh Thi Nguyen, Son Truong Nguyen, Mohd Said Nurumal, Aimable Nzabonimana, Nagla Abdelrahim Mohamed Ahmed Omer, Oluwabunmi Ogungbe, Angela Chiu Yin Poon, Areli Reséndiz-Rodriguez, Busayasachee Puang-Ngern, Ceryl G Sagun, Riyaz Ahmed Shaik, Nikhil Gauri Shankar, Kathrin Sommer, Edgardo Toro, Hanh Thi Hong Tran, Elvira L Urgel, Emmanuel Uwiringiyimana, Tita Vanichbuncha, Naglaa Youssef

**Affiliations:** 1 School of Nursing, Li Ka Shing Faculty of Medicine, University of Hong Kong, Hong Kong SAR, China; 2 School of Nursing, Johns Hopkins University, Baltimore, Maryland, USA; 3 Vice-Chancellor and Principal, University of Wollongong, Wollongong, Australia; 4 School of nursing, The Hong Kong Polytechnic University, Hong Kong SAR, China; 5 School of Nursing and Health Studies, Hong Kong Metropolitan University, Hong Kong SAR, China; 6 Department of Social Work, East China University of Science and Technology, Shanghai, China; 7 School of Nursing, The University of British Columbia, Kelowna British Columbia, Canada; 8 The Nethersole School of Nursing, The Chinese University of Hong Kong, Hong Kong; 9 Institute of Nursing Research, Osogbo, Osun State, Nigeria; 10 College of Medicine, Majmaah University, Al Majmaah, Saudi Arabia; 11 Department of Family & Community Medicine, College of Medicine, Majmaah University, Majmaah, Saudi Arabia; 12 Department of Internal Medicine, Al-Azhar University, Cairo, Egypt; 13 Department of Urology, College of Medicine, Majmaah University, Al Majmaah, Saudi Arabia; 14 School of Public Health, JSS Medical College, JSS AHER, Mysuru, India; 15 Department of Statistics, Chulalongkorn Business School, Bangkok, Thailand; 16 Kulliyyah of Nursing, International Islamic University, Kuantan, Malaysia; 17 Italian Association against Leukemia, Lymphoma and Myeloma, Rome Section, Rome, Italy; 18 Diálogos Guatemala, Guatemala, Guatemala; 19 School of Nursing, Centro Escolar University, Manila, Philippines; 20 Nursing Department, Faculty of Health Science, Beirut Arab University, Beirut, Lebanon; 21 Italian Group for Adult Hematologic Disease, Data Center and Health Outcomes Research Unit, Rome, Italy; 22 Department of Psychology, Beirut Arab University, Beirut, Lebanon; 23 Department of hepatogastroenterology and infectious diseases, Damietta faculty of medicine, Al-Azher University, Cairo, Egypt; 24 Ergonomics Research Center, University of Guadalajara, Jalisco, Mexico; 25 Laboratory of Applied Prosocial Research, Department of Basic, Developmental and Educational Psychology, Autonomous University of Barcelona, Barcelona, Spain; 26 Faculty of Health Sciences, University of Lethbridge, Alberta, Canada; 27 Faculty of Nursing, Ladoke Akintola University of Technology, Ogbomosho, Nigeria; 28 Alice Lee Centre for Nursing Studies, National University of Singapore, Singapore; 29 School of Nursing, Wijaya Husada Health Institute, Bogor, Indonesia; 30 Department of Optometry, University of Kwazulu-Natal, Durban, South Africa; 31 Ecove, Ghaziabad, India; 32 School of Nursing, Paramedicine and Health Care Science, Charles Sturt University, New South Wales, Australia; 33 Nam Dinh University of Nursing, Nam Dinh, Vietnam; 34 Pontificia Universidad Católica de Valparaíso, School of Social Work, Valparaíso, Chile; 35 Research Department, National Commission for Medical Arbitration, Mexico City, Mexico; 36 College of Medicine and Health Sciences, University of Rwanda, Kigali, Rwanda; 37 Department of Physiotherapy, Faculty of Science and Technology, São Paulo State University (UNESP), Presidente Prudente, Brazil; 38 Pharmacology and Toxicology Department, Faculty of Pharmacy, Benghazi University, Benghazi, Libya; 39 School of Nursing and Midwifery, College of Medicine and Health Sciences, University of Rwanda, Kigali, Rwanda; 40 Center for Language Enhancement, College of Arts and Social Sciences, University of Rwanda, Huye, Rwanda; 41 Faculty of Medicine, Alzaiem Alazhari University, Khartoum North, Sudan; 42 Faculty of Health Sciences and Sports, Macao Polytechnic University, Macao, China; 43 National Autonomous University of Mexico, Mexico City, Mexico; 44 Mental Health and Learning division, Wrexham Maelor Hospital, Wrexham, UK; 45 Medical-surgical Nursing Department, Faculty of Nursing, Cairo University, Cairo, Egypt

## Abstract

**Background:**

Given the limited understanding of individuals’ positive gains, this study aimed to identify these gains that could be leveraged by policymakers to enhance future health and societal resilience.

**Methods:**

We used a global qualitative approach to survey adults over 18 from 30 countries across six World Health Organization (WHO) regions, who detailed up to three personal positive gains from COVID-19 pandemic via an open-ended question. Inductive thematic analysis was employed to identify main themes, and quantitative methods were used for demographic and regional comparisons based on the percentage of responses for each theme.

**Results:**

From 35 911 valid responses provided by 13 853 participants, six main themes (one negative theme), 39 subthemes, and 673 codes were identified. Five positive gain themes emerged, ordered by response frequency: 1) improved health awareness and practices; 2) strengthened social bonds and trust; 3) multi-dimensional personal growth; 4) resilience and preparedness building; 5) accelerated digital transformation. The percentage of responses under these themes consistently appeared in the same order across various demographic groups and economic development levels. However, there were variations in the predominant theme across WHO regions and countries, with either Theme 1, Theme 2, or Theme 3 having the highest percentage of responses. Although our study primarily focused on positive gains, unexpectedly, 12% of responses (4304) revealed ‘negative gains’, leading to an unforeseen theme: ‘Distrust and emerging vulnerabilities.’ While this deviates from our main topic, we retained it as it provides valuable insights. Notably, these ‘negative gains’ had a higher percentage of responses in areas like Burundi (94.1%), Rwanda (31.8%), Canada (26.9%), and in the African Region (37.7%) and low-income (43.9%) countries, as well as among non-binary individuals, those with lower education, and those facing employment challenges.

**Conclusions:**

Globally, the identified diverse positive gains guide the domains in which health policies and practices can transform these transient benefits into enduring improvements for a healthier, more resilient society. However, variations in thematic responses across demographics, countries, and regions highlights need for tailored health strategies.

The COVID-19 pandemic, an unprecedented global crisis, has indelibly reshaped all facets of life globally – health, economy, environment, and societal fabric [1–3]. Major societal events are often transformative. The predominant focus on the pandemic's negative impacts, necessitates a more balanced perspective to prevent a skewed understanding of its overall impacts. Additionally, unveiling the hidden positive outcomes or unintended consequences is essential for developing well-rounded strategies that sustain these shifts and foster hope and resilience.

In this study, positive gains refer to the diverse range of beneficial outcomes that individuals experienced as a result of the COVID-19 pandemic. The premise that adversity can lead to positive outcomes is supported by several theoretical perspectives [4–6]. First, Post-Traumatic Growth theory suggests that adversity can foster personal growth and resilience [4]. Second, Social Cognitive Theory emphasises that individual factors and behavioural adaptations can catalyse broader societal changes, especially in public health and social norms [5]. Lastly, Collective Behavior and Social Movements theory highlights the pandemic's potential to drive societal evolution and initiate new social movements [6]. These theories collectively underpin our expectation that, despite its challenges, the pandemic could yield positive developments.

Emerging literature, albeit limited, has begun to identify several positive outcomes of the pandemic, such as environmental benefits, strengthened relationships, new hobbies, improved hygiene, healthier lifestyles, and technological innovation [1,7–12]. However, these primarily quantitative studies have often been limited in scope, focusing on specific groups or regions. This leaves a gap in understanding the broader spectrum of the pandemic's positive gains, especially across different global contexts.

In response to the research gap, this study adopts a qualitative approach, leveraging open-ended questions to capture diverse and rich personal positive experiences globally. Our study strives to offer a comprehensive perspective on the pandemic's overlooked positive gains, informing policy and societal development strategies.

## Methods

### Methodological orientation

This study utilised an interpretative qualitative approach to identify themes in participant narratives about positive gains from the COVID-19 pandemic. This method was chosen for its direct insight derivation from qualitative data, aligning well with the study's exploratory nature.

### Participants and setting

In this cross-culture and cross-sectional study, we engaged individuals aged 18 and older from 30 countries using convenience sampling [13]. Data collection, spanning from July 2020 to August 2021and specifically targeting responses to the open-ended question, involved both digital methods (such as a dedicated survey website and social media) and traditional methods (offline surveys), thereby encompassing settings online and at various physical locations. Of 16 512 respondents, 2659 (16.1%) provided no response. The survey's design indicates independent responses from participants in the absence of non-participants.

### Data collection

In addition to completing demographic questions, participants were invited to share up to three positive outcomes they experienced during the COVID-19 pandemic. The instrument was an open-ended question: ‘What have you gained from the COVID-19 pandemic? Please list the top three gains.’

### Translation and data preparation

A reputable translation company was engaged to translate all non-English responses into English, thereby ensuring accuracy. Their approach encompassed translation by expert linguists, a comprehensive quadruple-check quality control process (translator, editor, engineer, and linguistic for checking), and the integration of client feedback, guaranteeing both linguistic accuracy and contextual fidelity. The translated English versions then served as the basis for thematic analysis. As an online survey limitation, translated transcripts were not returned to participants for feedback or corrections.

### Qualitative analysis

We used spreadsheets to analyse qualitative data, chosen based on the data type and participant responses [14]. An inductive thematic analysis following Braun and Clarke’s six-phase framework was applied, allowing themes to emerge naturally without predefined categories [15].

#### Coding process and coders

Primary coder JYL familiarised herself with the data by thoroughly reviewing all responses, identifying preliminary ideas and potential codes [15]. The coding process involved two primary coders: JYL and YQL. Specifically, JYL identified initial codes based on her thorough engagement with the data. To enhance reliability and minimise individual bias, YQL. checked whether the coding is acceptable of a random subset of 10% of the responses (3591 responses), balancing representativeness and manageability [14]. Discrepancies were resolved through discussions, with about 1.5% of responses requiring consensus.

To assess inter-rater reliability and account for YQL coding only a portion of responses, we utilised ChatGPT-4 as an auxiliary checker. We measured the inter-rater reliability between the primary coder (JYL) and ChatGPT-4 using percent agreement [16], considering ChatGPT-4's coding was dependent on JYL’s initial classifications. Among 35 911 codings, we observed a 94.5% agreement rate, demonstrating strong concordance between the coders.

#### Theme development and thematic saturation

JYL grouped similar codes into subthemes using a constant comparison method [17] and then iteratively integrated these into broader themes aligned with the research questions. She continuously assessed the coherence among responses, codes, subthemes, and themes, making adjustments to ensure the themes were data-derived and accurately reflected participants' experiences.

Thematic saturation was reached when no new themes or subthemes emerged from additional data, determined through continuous review and iterative coding [18]. Both coders identified saturation during the middle of the coding process, but continued coding all survey responses to facilitate quantitative comparison.

#### Defining and naming themes and producing report

After achieving thematic consistency, JYL precisely defined each theme and subtheme, aligning them with the research questions and supporting them with illustrative examples. Due to the online survey method, returning findings to participants for feedback was not feasible.

#### Research team and reflexivity

The first author (JYL) who led this study is a female researcher with experience in conducting qualitative research, focusing on the global impact of COVID-19 [1]. Her background provides a perspective on the pandemic's multifaceted impacts. Prior to the commencement of this study, JYL had no established relationships with the participants, who were unaware of her research goals and motivations. Her research experience in global COVID-19 impacts ensures an objective and well-informed perspective.

### Supplementary quantitative analysis

We further quantified our qualitative findings for cross-demographic, cross-country and regional comparisons. We first assessed the prevalence of 'negative gains', an unexpected outcome in our primarily 'positive gains' focused study. This involved calculating the proportion of negative responses in each group or region to gauge the extent of negative impacts and contextualise positive responses. Subsequently, we redefined 'positive gains' as our entire data set (100%) to analyse the proportion of each positive theme within all positive responses across different areas or groups. For regional comparisons, countries were categorised into six World Health Organization regions: African (AFR), European (EUR), South-East Asia (SEAR), Americas (AMR), Eastern Mediterranean (EMR), and Western Pacific (WPR). They were also classified by economic development levels into low-income countries (LICs), lower-middle-income countries (LMICs), upper-middle-income countries (UMICs), and high-income countries (HICs), following the World Bank's 2020 classification [1].

### Ethics approval

The institutional review board of the University of Hong Kong-the Hospital Authority Hong Kong West Cluster approved this study, reference no: UW 20–272. This study obtained informed consent from all participants and ensured anonymity by not collecting identifiable personal information. All responses were anonymised and securely stored with restricted access. Participants were informed that participation was voluntary and could withdraw at any time if they feel emotional distress. These measures prioritised data security and participant well-being throughout the research process.

## Results

### Participants’ sociodemographics and geographical distribution

**Figure 1:**
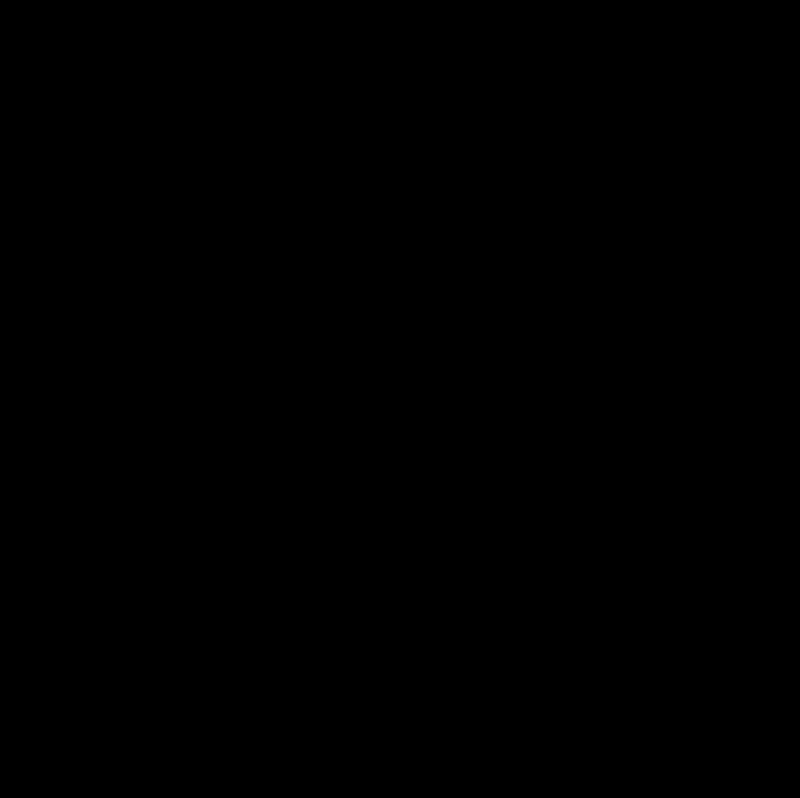
Flowchart of data cleaning.

In our international sample of 16 512 adults, 13 853 participants provided at least one response. This group comprised 9214 young adults (66.5%), 3519 middle adults (25.4%), and 1120 older adults (8.1%). The gender distribution included 8827 females (63.7%), 4940 males (35.7%), and 86 non-binary individuals (0.6%). **Table 1** provides additional socio-demographic details and geographical distribution. After cleaning data for all received responses, we obtained 35 911 valid responses for subsequent thematic analysis (**Figure 1**).

**Table 1:** Sociodemographics, country, and region details of participants and their thematic responses distribution (n = 13 853)

Groups	No. of participants (% of total)	Total responses	Response in Theme 6* (% in total response)	Total positive responses	No. of responses (% in total positive responses)				
					**Theme 1†**	**Theme 2‡**	**Theme 3§**	**Theme 4¶**	**Theme 5║**
**Sociodemographics**									
Age									
*Young adult (<35 y)*	9214 (66.5)	24096	2876 (11.9)	21220	8198 (38.6)	4518 (21.3)	5020 (23.7)	2540 (12.0)	944 (4.4)
*Middle adult (35–60 y)*	3519 (25.4)	9005	1128 (12.5)	7877	3109 (39.5)	2011 (25.5)	1555 (19.7)	883 (11.2)	319 (4.0)
*Older adult (>60 y)*	1120 (8.1)	2810	300 (10.7)	2510	1106 (44.1)	594 (23.7)	471 (18.8)	258 (10.3)	81 (3.2)
Gender									
*Female*	8827 (63.7)	23206	2542 (11.0)	20664	7987 (38.7)	4853 (23.5)	4647 (22.5)	2297 (11.1)	880 (4.3)
*Male*	4940 (35.7)	12471	1701 (13.6)	10770	4358 (40.5)	2237 (20.8)	2357 (21.9)	1359 (12.6)	459 (4.3)
*Non-binary*	86 (0.6)	234	61 (26.1)	173	68 (39.3)	33 (19.1)	42 (24.3)	25 (14.5)	5 (2.9)
Marital status									
*Single*	7246 (52.3)	19079	2125 (11.1)	16954	6515 (38.4)	3375 (19.9)	4231 (25.0)	2079 (12.3)	754 (4.4)
*Separated/divorced/widowed*	559 (4.03)	1421	168 (11.8)	1253	453 (36.2)	295 (23.5)	296 (23.6)	165 (13.2)	44 (3.5)
*Married/cohabitation/common-law*	6048 (43.7)	15411	2011 (13.0)	13400	5445 (40.6)	3453 (25.8)	2519 (18.8)	1437 (10.7)	546 (4.1)
Education									
*Primary or below*	323 (2.3)	811	408 (50.3)	403	185 (45.9)	117 (29.0)	58 (14.4)	41 (10.2)	2 (0.5)
*Secondary*	1829 (13.2)	4281	821 (19.2)	3460	1435 (41.5)	899 (26.0)	731 (21.1)	294 (8.5)	101 (2.9)
*Associate degree*	1271 (9.2)	3234	399 (12.3)	2835	1317 (46.5)	585 (20.6)	521 (18.4)	304 (10.7)	108 (3.8)
*College*	1811 (13.1)	4587	574 (12.5)	4013	1553 (38.7)	929 (23.1)	952 (23.7)	433 (10.8)	146 (3.6)
*Bachelor*	5772 (41.7)	15217	1477 (9.7)	13740	5579 (40.6)	2853 (20.8)	3163 (23.0)	1596 (11.6)	549 (4.0)
*Graduate*	2686 (19.4)	7331	602 (8.2)	6729	2238 (33.3)	1599 (23.8)	1509 (22.4)	980 (14.6)	403 (6.0)
*Missing value*	161 (1.2)	450	23 (5.1)	427	106 (24.8)	141 (33.0)	112 (26.2)	33 (7.7)	35 (8.2)
Occupation									
*Job seeking*	715 (5.2)	1761	264 (15.0)	1497	609 (40.7)	309 (20.6)	357 (23.8)	156 (10.4)	66 (4.4)
*Laid off*	103 (0.7)	237	73 (30.8)	164	75 (45.7)	39 (23.8)	32 (19.5)	16 (9.8)	2 (1.2)
*Not in workforce*	797 (5.8)	1923	227 (11.8)	1696	772 (45.5)	434 (25.6)	305 (18.0)	146 (8.6)	39 (2.3)
*Retired*	523 (3.8)	1347	159 (11.8)	1188	513 (43.2)	283 (23.8)	220 (18.5)	136 (11.4)	36 (3.0)
*Self-employed*	1178 (8.5)	3114	885 (28.4)	2229	883 (39.6)	478 (21.4)	471 (21.1)	298 (13.4)	99 (4.4)
*Student*	3973 (28.7)	10818	1132 (10.5)	9686	3677 (38.0)	2050 (21.2)	2595 (26.8)	960 (9.9)	404 (4.2)
*Working (1–39 h/week)*	2163 (15.6)	5568	528 (9.5)	5040	1936 (38.4)	1188 (23.6)	1065 (21.1)	638 (12.7)	213 (4.2)
*Working (> = 40 h/week)*	4401 (31.8)	11143	1036 (9.3)	10107	3948 (39.1)	2342 (23.2)	2001 (19.8)	1331 (13.2)	485 (4.8)
Confirmed with COVID-19									
*Yes*	466 (3.4)	1247	128 (0.1)	1119	312 (27.9)	275 (24.6)	333 (29.8)	162 (14.5)	37 (3.3)
*No*	13387 (96.6)	34664	4176 (0.1)	30488	12101 (39.7)	6848 (22.5)	6713 (22.0)	3519 (11.5)	1307 (4.3)
Country stay									
*Australia*	160 (1.2)	371	44 (11.9)	327	105 (32.1)	89 (27.2)	73 (22.3)	40 (12.2)	20 (6.1)
*Brazil*	466 (3.4)	1289	287 (22.3)	1002	205 (20.5)	296 (29.5)	306 (30.5)	145 (14.5)	50 (5.0)
*Burundi*	361 (2.6)	1028	967 (94.1)	61	45 (73.8)	2 (3.3)	6 (9.8)	8 (13.1)	0 (0.0)
*Canada*	319 (2.3)	850	229 (26.9)	621	153 (24.6)	196 (31.6)	144 (23.2)	81 (13.0)	47 (7.6)
*Chile*	290 (2.1)	786	23 (2.9)	763	156 (20.4)	281 (36.8)	193 (25.3)	90 (11.8)	43 (5.6)
*Egypt*	457 (3.3)	1174	82 (7.0)	1092	491 (45.0)	162 (14.8)	282 (25.8)	103 (9.4)	54 (4.9)
*Guatemala*	186 (1.3)	491	10 (2.0)	481	91 (18.9)	143 (29.7)	165 (34.3)	52 (10.8)	30 (6.2)
*Hong Kong*	1470 (10.6)	2945	123 (4.2)	2822	1362 (48.3)	795 (28.2)	377 (13.4)	249 (8.8)	39 (1.4)
*India*	356 (2.6)	940	61 (6.5)	879	308 (35.0)	240 (27.3)	202 (23.0)	102 (11.6)	27 (3.1)
*Indonesia*	482 (3.5)	1421	151 (10.6)	1270	753 (59.3)	176 (13.9)	186 (14.6)	87 (6.9)	68 (5.4)
*Italy*	186 (1.3)	530	69 (13.0)	461	146 (31.7)	132 (28.6)	101 (21.9)	66 (14.3)	16 (3.5)
*Lebanon*	440 (3.2)	1127	87 (7.7)	1040	446 (42.9)	213 (20.5)	222 (21.3)	114 (11.0)	45 (4.3)
*Libya*	532 (3.8)	1387	122 (8.8)	1265	563 (44.5)	226 (17.9)	341 (27.0)	96 (7.6)	39 (3.1)
*Macau*	204 (1.5)	552	17 (3.1)	535	222 (41.5)	134 (25.0)	116 (21.7)	50 (9.3)	13 (2.4)
*Mainland China*	569 (4.1)	1544	94 (6.1)	1450	449 (31.0)	461 (31.8)	395 (27.2)	114 (7.9)	31 (2.1)
*Malaysia*	493 (3.6)	1424	123 (8.6)	1301	572 (44.0)	277 (21.3)	233 (17.9)	161 (12.4)	58 (4.5)
*Mexico*	785 (5.7)	2063	83 (4.0)	1980	429 (21.7)	541 (27.3)	640 (32.3)	275 (13.9)	95 (4.8)
*Nigeria*	463 (3.3)	1218	38 (3.1)	1180	456 (38.6)	133 (11.3)	335 (28.4)	174 (14.7)	82 (6.9)
*Philippines*	428 (3.1)	1232	137 (11.1)	1095	426 (38.9)	244 (22.3)	267 (24.4)	135 (12.3)	23 (2.1)
*Republic Of Sudan*	476 (3.4)	1282	93 (7.3)	1189	514 (43.2)	171 (14.4)	335 (28.2)	133 (11.2)	36 (3.0)
*Rwanda*	137 (1.0)	390	124 (31.8)	266	122 (45.9)	35 (13.2)	41 (15.4)	53 (19.9)	15 (5.6)
*Saudi Arabia*	519 (3.8)	1408	88 (6.3)	1320	482 (36.5)	262 (19.8)	372 (28.2)	154 (11.7)	50 (3.8)
*Singapore*	213 (1.5)	609	27 (4.4)	582	158 (27.1)	170 (29.2)	139 (23.9)	76 (13.1)	39 (6.7)
*South Africa*	173 (1.3)	463	40 (8.6)	423	111 (26.2)	117 (27.7)	125 (29.6)	52 (12.3)	18 (4.3)
*South Korea*	2238 (16.2)	5322	851 (16.0)	4471	2249 (50.3)	858 (19.2)	807 (18.0)	384 (8.6)	173 (3.9)
*Spain*	44 (0.3)	108	3 (2.8)	105	19 (18.1)	26 (24.8)	38 (36.2)	15 (14.3)	7 (6.7)
*Thailand*	690 (5.0)	1989	183 (9.2)	1806	743 (41.1)	241 (13.3)	170 (9.4)	497 (27.5)	155 (8.6)
*UK*	187 (1.4)	500	55 (11.0)	445	108 (24.3)	120 (27.0)	148 (33.3)	51 (11.5)	18 (4.0)
*USA*	198 (1.4)	549	42 (7.7)	507	127 (25.0)	177 (34.9)	120 (23.7)	49 (9.7)	34 (6.7)
*Vietnam*	331 (2.4)	919	51 (5.5)	868	402 (46.3)	205 (23.6)	167 (19.2)	75 (8.6)	19 (2.2)
World Health Organization region									
*African Region*	1134 (8.2)	3099	1169 (37.7)	1930	734 (38.0)	287 (14.9)	507 (26.3)	287 (14.9)	115 (6.0)
*Region of the Americas*	2244 (16.2)	6028	674 (11.2)	5354	1161 (21.7)	1634 (30.5)	1568 (29.3)	692 (12.9)	299 (5.6)
*Eastern Mediterranean Region*	2424 (17.5)	6378	472 (7.4)	5906	2496 (42.3)	1034 (17.5)	1552 (26.3)	600 (10.2)	224 (3.8)
*European Region*	417 (3.0)	1138	127 (11.2)	1011	273 (27.0)	278 (27.5)	287 (28.4)	132 (13.1)	41 (4.1)
*South-East Asia Region*	1528 (11.0)	4350	395 (9.1)	3955	1804 (45.6)	657 (16.6)	558 (14.1)	686 (17.3)	250 (6.3)
*Western Pacific Region*	6106 (44.1)	14918	1467 (9.8)	13451	5945 (44.2)	3233 (24.0)	2574 (19.1)	1284 (9.5)	415 (3.1)
Economic development									
*Low-income countries*	974 (7.0)	2700	1184 (43.9)	1516	681 (44.9)	208 (13.7)	382 (25.2)	194 (12.8)	51 (3.4)
*Lower-middle-income countries*	2517 (18.2)	6904	520 (7.5)	6384	2836 (44.4)	1160 (18.2)	1439 (22.5)	676 (10.6)	273 (4.3)
*Upper-middle-income countries*	4334 (31.3)	11777	1029 (8.7)	10748	3609 (33.6)	2515 (23.4)	2597 (24.2)	1506 (14.0)	521 (4.8)
*High-income countries*	6028 (43.5)	14530	1571 (10.8)	12959	5287 (40.8)	3240 (25.0)	2628 (20.3)	1305 (10.1)	499 (3.9)

*Theme 6: distrust and emerging vulnerabilities.

†Theme 1: improved health awareness and practices.

‡Theme 2: strengthened social bonds and trust.

§Theme 3: multi-dimensional personal growth.

¶Theme 4: resilience and preparedness building.

║Theme 5: accelerated digital transformation.

### Qualitative findings

Thematic analysis yielded five main positive themes and an unexpected negative theme, 39 subthemes, and 673 codes emerged. The identified themes were:

1) improved health awareness and practices;

2) strengthened social bonds and trust;

3) multi-dimensional personal growth;

4) resilience and preparedness building;

5) accelerated digital transformation;

6) distrust and emerging vulnerabilities (this unexpected theme deviates from our main topic but was retained as it provides valuable insights).

**Table 2** outlines the structure of themes and subthemes and representative quotations.

**Table 2:** Overview of main themes, subthemes, and representative quotations

Main themes (response frequency)	Subtheme (response frequency)	Example quotations
Improved health awareness and practices (12 413)	Subtheme 1: increased health knowledge, awareness and practice (8214)	More knowledge from extra courses offered to health care professionals to upgrade skills.
		Knowing the methods of good prevention of diseases and knowing the beneficial and enhanced foods for health and immunity.
		Money is not everything, without health, no matter how much you earn, it is useless.
		Learning about health knowledge and practicing self-protection by wearing masks and staying home.
		More appreciation of mental health.
		Receiving correct information, such as health and hygiene knowledge, online.
	Subtheme 2: health behaviour adaptations and lifestyle changes (2139)	Establishing a regular schedule, healthy diet, and increasing exercise.
		More time to focus on my diet and health, I have lost 10lbs.
		Frequent exercise and drink plenty of vitamins so that your metabolism goes well.
		More regular sleeping schedule.
		I stopped drinking.
		Gave up smoking cigarettes.
	Subtheme 3: self-renewal through rest and recuperation (938)	Being able to slow down and do things you enjoy.
		Less time commuting to work, more time do get things done at home.
		Relaxation from the pressures of social and work life.
		Rest and recuperation at home, which we've been missing because of my long shift all week.
		How nice it is not to waste time in traffic on the way/back from work.
		Less time worrying about everyone's schedules and who needs to be where when.
		Spare time to contemplate life matters and slow down the pace of life.
	Subtheme 4: rediscovering home: enhancement and living well-being (671)	A much-appreciated opportunity to focus and spend time on activities within the home environment as not going out as much.
		The importance of designing the home environment to be more comfortable and have appropriate facilities for various health, sports, and family activities.
		Order my house and feel comfortable in it.
		Having time to put a little more order at home, getting rid of objects that were no longer used.
	Subtheme 5: eco-conscious global well-being (251)	It's very scary, we must protect wildlife, do not eat wild animals indiscriminately, each of us must protect ourselves and cherish nature.
		Need to look after environment as get clean after stopped pollution due to covid.
		The importance of nature and association of man with the nature.
		Fresher air.
	Subtheme 6: direct enhancements in holistic health (200)	Psychological comfort away from problems and worries.
		Better mental management.
		Enjoyment in life.
		Improve the life quality.
		My skin has improved due to lack of going out in the sun.
Strengthened social bonds and trust (7123)	Subtheme 7: strengthened relationships and interactions (5553)	Reconciled with my immediate family members. We communicate more often and don't fight as much. We try not to take each other for granted, as we have lost many extended family members to COVID-19.
		Closer ties with family and friends, albeit online.
		Increasing family cohesion, perhaps to the degree of knowing what you are thinking without talking.
		More time for teaching children and accompanying their growth.
		I played more with my puppy.
		Getting to know more neighbors and helping each other.
	Subtheme 8: social responsibility and community engagement (1192)	Engaging in some anti-pandemic activities.
		Important of teamwork to handle this situation starting from citizen up to government.
		The teamwork between the netizen to stay at home, sanitize, wash hands, wear mask and distance themselves is very very important.
		Providing generosity to vulnerable groups in society during the pandemic is the right thing to do.
		Unity in action ... like wearing a mask ... will help us all get through it; we need each other.
		Greater awareness of how communities need to work together and look after each other.
	Subtheme 9: trust in government and society and value the health systems (378)	Understanding how proud and privileged it is to live in a strong and loving nation.
		We can only fight it if we adhere to the government strategy.
		The people of the whole country actively respond to the call of the nation, fight against the epidemic. The nation loves the people, and the people love the nation. The Communist Party serves the people, and the people support the leadership of the Party.
		Strive to study and lay a foundation for better development of the country, society, and individuals in the future.
		Appreciate all the health care worker for their hard work, and all the front lines for their efforts.
		Government management has a huge impact against the spread of disease.
		Increase my respect for the medical staff who stood in the face of the crisis.
Multi-dimensional personal growth (7046)	Subtheme 10: Transformative inner landscapes: emotional and philosophical realignment (2406)	Understanding the impermanence of life and being grateful for good health.
		I felt the strength and the power of God and the weakness of the whole world stopped dead virus.
		I learned to value life, health and the people around me more.
		Discover aspects of yourself that have never been discovered before.
		I have gained a deeper understanding of the meaning of life.
		It made me more aware of my thoughts and emotions.
		It helps some people discover hidden talent they wouldn't have discovered if not for the pandemic.
		Live and enjoy small daily joys.
		Nothing forever.
		Humanity.
		Life is short.... Just enjoy.
		I knew the value of everything I own and that money does not answer all that its prophet.
		Life is very uncertain.
		There are so many material things and activities in life that we can truly do without!
		Money and social life are not everything in this life.
	Subtheme 11: time resourcefulness, allocation, and value-added living (1265)	More time on having the things I don’t usually do during normal days because of busy schedule.
		Being able to have recreation time with audiovisual productions.
		Learned to priorities things that are important.
		You taught me how to organize my free time with something useful to me.
		Knowing how to take advantage of the time that before would have thought was wasted.
		Having more time for myself.
	Subtheme 12: mindful leisure, skills development and hobbies (1056)	Skill. Because I had time, I was able to develop my coding, animation and photoshopping skills.
		Take advantage of leisure time by reading and discovering new hobbies.
		Focus on learning new languages, getting to know different cultures, etc.
	Subtheme 13: dedication, perseverance and growth mindset (807)	Trying my best to be productive during the most trying times.
		More intentionality on what to pursue.
		Focus on self-development.
		Never stop learning new things to keep up with the era of disruption, otherwise, the world will change us. It's never too late for us to study.
		Sense of accomplishment in my contributions.
		Be wiser and more focused in my plans.
		Focus and hard work makes you stronger.
	Subtheme 14: Academic/career progression and development (802)	We have a lot of research to still carry out as regards health.
		Time to focus on my academic responsibilities.
		I was distinguished academically because of my studies in the quarantine period.
		Acquire new knowledge and certificates online.
		Opportunity to work in ICU.
	Subtheme 15: personal empowerment and autonomy (612)	Self-discipline, striving to become excellent and useful to society.
		Learning how to be self-sufficient.
		Confidence in facing rising challenge.
		Discovering your sovereignty and taking responsibility for yourself and what you can control.
		I can alone handle my things and stop depending on others.
		Realization that I have the power to change things.
	Subtheme 16: embracing simplicity and minimalism (98)	You can live in the simplest and least complicated way.
		Most of what we get is surplus, not indispensable goods.
		We can live without a lot of stuff.
		Minimalist life.
Resilience and preparedness building (3681)	Subtheme 17: acceptance, coping, and resilience (1066)	Realizing the uncertainty of life, accidents can happen anywhere, and feeling more calm.
		Mindset to not give up, but continue and 'make lemonade out of lemons'.
		We must have faith and patience.
		Control of emotions, mind, and anxiety should be taken care of.
		Resignation to circumstances and calm down accordingly.
		I know that I have mental strength and I am resilient.
		Meditation, paying respect to the Buddha, Calming your mind.
		Accept the change.
		Being resilient.
	Subtheme 18: financial dynamics and management (1042)	More money saved from not going out for expenses.
		The importance of getting prepare in term of emergency fund.
		The importance of having a source of income other than employment.
		Personal and business financial planning to be able to get through the Covid situation well.
	Subtheme 19: Job maintenance or new job opportunities (390)	Marketing and earning money at home through the Internet and delivery service.
		Having lots of free time and starting my own business.
		Keep my job.
		New Telehealth job opportunity.
		Found several part-time jobs.
	Subtheme 20: planning and preparedness (339)	Stocking medical supplies.
		Always have a backup plan.
		Preparedness: temporal/spiritual needs.
		It teaches us how to be prepared next time something bad happen like pandemic etc.
		Planning the medium and long-term future to be ready for the challenges already known, environment, education, science above all.
		Life management and planning including problem solving.
		Preparing for unexpected events is very important (e.g. reserves, health insurance).
	Subtheme 21: adaptation to change and flexibility (309)	Adapting to changes.
		Plan changes.
		Adjusting the pace of life.
		How to survive at home.
		Adjust your thoughts and expectations to be flexible with the situation.
		Be flexible, i.e. adapt to changes easily.
	Subtheme 22: learning and growth from experience and new opportunities (192)	All things happened have their own lesson back then.
		The knowledge that there are a vast amount of untapped opportunities to make progress.
		Every challenge is an opportunity to grow and increase.
		Opportunities always present themselves in difficult times.
		Experience gained crisis to work in difficult conditions.
		More to learn from experience and insight into how humanity.
	Subtheme 23: governmental aid in crisis and challenge (181)	10 000 HKD.
		Government's consumption card.
		Supplies such as masks, food, daily necessities, etc...
		Can withdraw The Employees Provident Fund (EPF) from account number 2.
		Anti-epidemic fund assistance.
	Subtheme 24: crisis and challenge response (162)	Ability to deal with emergencies.
		Risk management.
		Improved information gathering skills.
		Survival instincts.
		Dealing with crises.
		Finding alternative solutions to the conduct of life.
Accelerated digital transformation (1344)	Subtheme 25: online learning and digital transformation (421)	Learning online courses.
		Useful courses online.
		Learning methods for online courses.
		Physical presence is not a barrier to learning as other online means worked effectively.
	Subtheme 26: remote work and digital transformation (342)	Hold meetings remotely.
		Work from home successful idea.
		Remote working effectively.
		Participate in many workshops, research and online training courses.
		I am doing my professional practice via teleworking.
	Subtheme 27: digital awareness, literacy and skill development (264)	Greater computer literacy and confidence online.
		Managing banking online.
		Increased knowledge of the internet.
		Virtual and ICT skills are important.
	Subtheme 28: Enhanced digital communication and social (193)	Making more online friends.
		Greater remote connection with friends.
		Importance of online communication platform.
		Communication is possible electronically.
	Subtheme 29: Online shopping and digital transformation (57)	Online shopping trust.
		The convenience of online shopping.
		Increased use of online grocery shopping and delivery.
	Subtheme 30: e-commerce and digital transformation (51)	Constant internet usage served as a tool that grew my business during the pandemic. Before the pandemic, I focused on my offline clients more.
		Businesses should support digital channels.
		App Delivery.
	Subtheme 31: remote health care and digital transformation (16)	Access to telephone medical consultations.
		Better access to psychotherapy through telehealth.
		Online health care access.
Distrust and emerging vulnerabilities (4304)	Subtheme 32: emotional challenges & struggles (1367)	Anxiety, stress, depression, fear, worry, boredom, loneliness.
		I gained nothing... Almost lost everything.
	Subtheme 33: unmet basic needs (860)	Poverty, hunger, theft, unrest.
		It changed the lives of many, it brought poverty.
		Loss of rights.
	Subtheme 34: distrust in government, health system, media, and society (587)	Now I see how health systems are corrupted along with media and politicians.
		Politicians only care about their own interests.
		The poverty of the whole world mentally, materially, and morally.
		Healthcare decisions can really, really be affected by political interest.
		Unclear and ineffective government measures.
		Distrust in mainstream media and health intuitions.
		A lot of fake news.
	Subtheme 35: declined physical health (530)	Weight gain, illness, hypochondriasis.
		Skin problems caused by the mask.
		A disease that got severe due to lack of non-covid health care.
	Subtheme 36: restrictions and challenges in social and daily life (392)	Separation from loved ones.
		Lack of activity outside the home due to pandemic.
		Less travel.
		Disconnection with society.
		How hard is it not to see people's smiles or grimaces.
	Subtheme 37: financial difficulties and economic recession (288)	Economic crisis, financial problem, bankruptcy.
		More expensive things but less income.
		Unfortunately, taxes were imposed with the Corona crisis, which increased anxiety and psychological fatigue instead of alleviating the psychological crisis of the citizen.
	Subtheme 38: unemployment and career instability (217)	Lack of jobs.
		Loss of jobs.
		A career that was once thought to be stable is no longer the same. Have to look for other ways.
		More worry about job and future.
	Subtheme 39: education disruption and challenges (63)	Cannot going back to campus.
		Say no to studying abroad.
		School closures.
		Cannot lecture offline.

#### Improved health awareness and practices (Theme 1)

This theme describes the unexpected benefits of the COVID-19 pandemic on people’s health aspects worldwide. It highlights how the crisis has improved health awareness, knowledge, and habits at individual and environmental levels. At the individual level, people have become more health-conscious, prioritising well-being and learning about health risks (**Table 2**). Participants reported adopting improved hygiene and preventive health measures, along with healthier lifestyles that include more exercise and a healthy diet (**Table 2**). These changes also extend to personal spaces, as many become aware of and improve their home environments (**Table 2**). The pandemic has encouraged a slower pace of life, promoting self-renewal and overall health (**Table 2**). Additionally, while reducing environmental pollution, it has heightened awareness of the interconnectedness of health and the environment (**Table 2**). The perceived positive changes led to health improvements (**Table 2**), such as better sleep and weight loss. Overall, it reveals a significant shift in health consciousness and practices, leading to improved personal and environmental well-being during the pandemic.

#### Strengthened social bonds and trust (Theme 2)

This theme describes how the COVID-19 pandemic has positively intensified interpersonal connections, community cohesion, social accountability, and trust in societal systems. People have deepened bonds with family, friends, pets, and others (**Table 2**). Communities have seen a revival of altruism and civic engagement (**Table 2**). Trust in society, authorities, health systems, and governments has been renewed (**Table 2**). This theme emphasises the critical importance of social unity and shared responsibility in overcoming collective challenges and recognises these strengthened social ties as one of the pandemic's positive outcomes.

#### Multi-dimensional personal growth (Theme 3)

Theme 3 describes the multifaceted personal growth triggered by the pandemic, spanning both tangible and intangible realms. The intangible dimension is marked by a profound emotional and philosophical realignment (**Table 2**), enhancing self-understanding and reshaping value systems. Concurrently, there was an increased emphasis on personal empowerment and autonomy, coupled with a shift towards simplicity and minimalism (**Table 2**). In the tangible domain, notable strides were made in time management and value-added activities (**Table 2**), facilitating both personal and professional enrichment. This is further reflected in significant academic and career progressions, and the adoption of new skills and hobbies, alongside mindful leisure pursuits (**Table 2**). Together, these interwoven intangible and tangible elements underscore a holistic evolution in personal development during an unprecedented global challenge.

#### Resilience and preparedness building (Theme 4)

This theme explores the transformative journey of adaptation and resilience propelled by the COVID-19 pandemic, emphasising evolution through adversity. It highlights a spectrum of psychological responses such as acceptance, coping, and resilience (**Table 2**). It reveals how individuals and communities adaptively responded, showing flexibility amid altered life routines, and effectively tackled various crises and challenges (**Table 2**). A proactive approach was also evident in financial management and employment adaptability, including job retention and exploring new opportunities (**Table 2**). The emphasis on planning and preparedness (**Table 2**) highlights a forward-thinking response to the crisis. The pandemic period also fostered significant learning and growth from the adversity (**Table 2**), signifying a transformative shift from adversity to resilient development. Governmental aid (**Table 2**) played a supporting role in this transformation. Overall, this theme captures not only the immediate handling of crises but also the extensive learning and preparation for future challenges.

#### Accelerated digital transformations (Theme 5)

Theme 5 emphasises the swift shift to digital platforms prompted by the COVID-19 pandemic. It intricately maps the expansion across various sectors: online learning's surge (, the widespread adoption of remote work (, and the significant increase in digital literacy and skills (**Table 2**). The theme further explores the evolution of digital communication and social engagement, alongside the growth of online shopping and e-commerce (**Table 2**). Additionally, it highlights the emergence of remote health care (**Table 2**) as a new norm. This digital transformation illustrates how society swiftly embraced technology for education, work, commerce, and health care, showcasing technology's critical role in ensuring continuity and flexibility.

#### Distrust and emerging vulnerabilities (Theme 6)

Theme 6, which emerged unexpectedly from the request for the description of positive changes, reflects the multitude of challenges unleashed by the COVID-19 pandemic and potentially the paradox of emotions This response reflects the physical and emotional aspects of the pandemic (**Table 2**). This period was marked by a notable decline in public trust, with increased scepticism towards societal systems and authorities, underscoring a deep-rooted distrust in institutions (**Table 2**). The crisis also brought about social instability, manifesting in financial stress, job market turbulence, daily life constraints, educational disruptions , and fundamental survival challenges (**Table 2**). This narrative emphasises the urgent need for comprehensive health policies to tackle these varied impacts and restore trust in the aftermath of the pandemic.

### Quantitative findings

Globally, although our study initially targeted positive gains, we found that 12.0% (4304 out of 35 911) of responses reported negative gains, which fall under Theme 6. **Table 2** displays the number of responses for 6 themes as well as their subthemes.

#### Demographics disparities

For positive gains, the percentage of responses decreased from Theme 1 (highest) to Theme 5 (lowest) consistently across all demographic groups (Figure S1 in the **Online Supplementary Document**). Conversely, individuals identifying as non-binary, with lower education levels, or facing employment challenges such as self-employment or layoffs, reported more proportion of negative gains (Theme 6) (Figure S1 in the **Online Supplementary Document**).

#### National level

**Figure 2:**
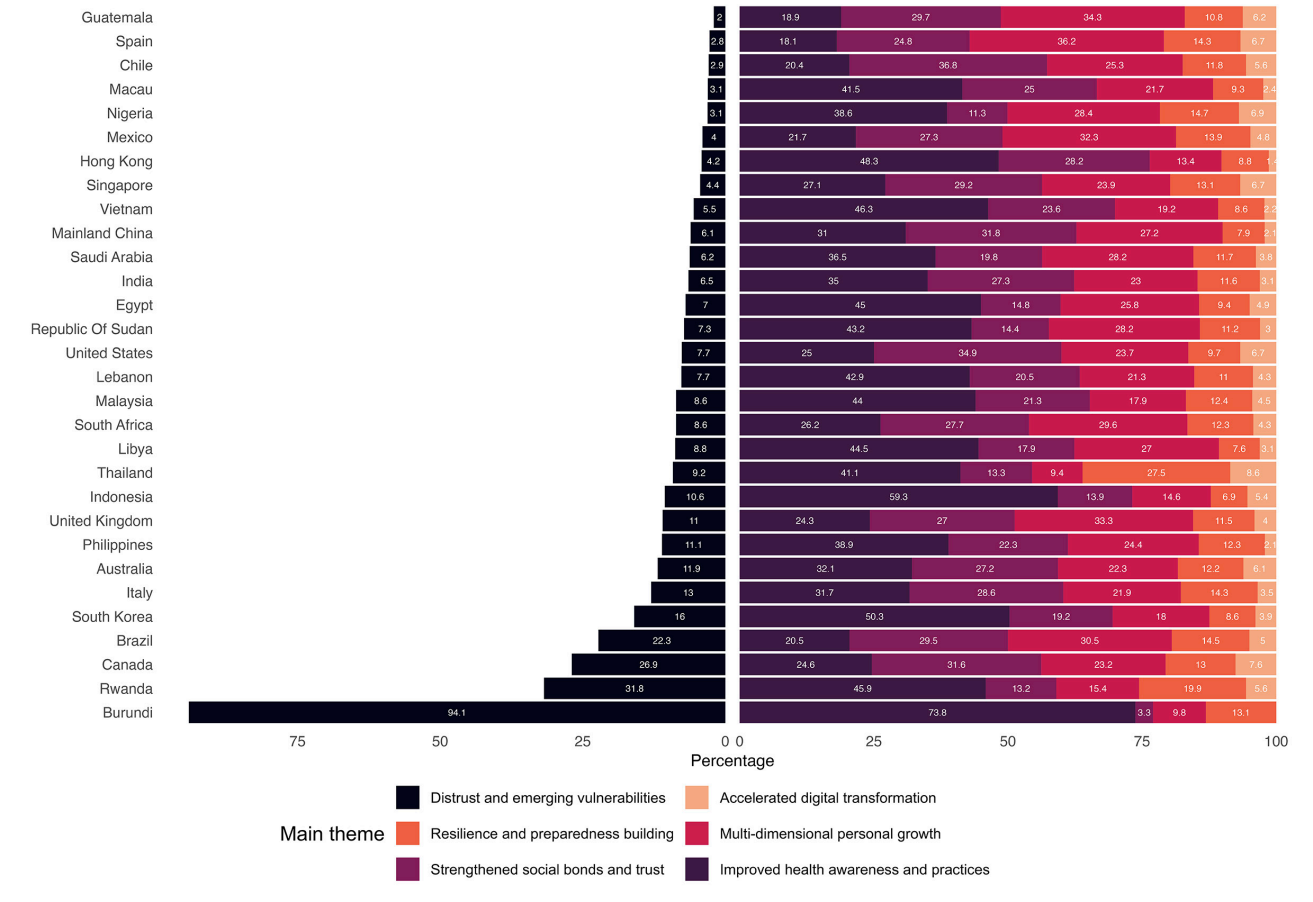
Proportional distribution of negative to all responses and positive by theme to all positive, by country (n = 35 911).

At the national level, the distribution of responses under Theme 1 varied from 18.1% in Spain to 73.8% in Burundi. For Theme 2, the proportions ranged from 3.3% in Burundi to 36.8% in Chile, and for Theme 3, they spanned from 9.4% in Thailand to 36.2% in Spain. Responses in Theme 4 were reported between 6.9% in Indonesia and 27.5% in Thailand, while Theme 5 responses varied from 0% in Burundi to 8.6% in Thailand. Notably, Theme 6 (negative gains) was most prevalent in Burundi (94.1%), Rwanda (31.8%), and Canada (26.9%), with the lowest percentages in Guatemala (2%), Spain (2.8%), and Chile (2.9%). Additionally, the thematic dominance of positive gains varied across nations. 19 countries, including Australia, Burundi, Egypt, Hong Kong, India, Indonesia, Italy, Lebanon, Libya, Macau, Malaysia, Nigeria, Philippines, Republic of Sudan, Rwanda, Saudi Arabia, South Korea, Thailand, and Vietnam, primarily reported gains in Theme 1, with the proportions ranging from 31.7% in Italy to 73.8% in Burundi. Canada, Chile, China, Singapore, and the United States reported primarily Theme 2 gains, with a range from 29.2% in Brazil to 36.8% in Chile. Theme 3 gains were notably prevalent in Brazil, Guatemala, Mexico, South Africa, Spain, and the UK, with proportions between 29.6% in South Africa and 36.2% in Spain. Notably, no countries reported predominant gains in Themes 4 and 5 (**Figure 2**).

#### Regional level

At the regional level, the distribution of responses for Themes 1 to 5 showed a range of proportions: 21.7% in the AMR to 45.6% in SEAR for Theme 1, 14.9% in AFR to 30.5% in AMR for Theme 2, 14.1% in SEAR to 29.3% in AMR for Theme 3, 9.5% in the WPR to 17.3% in SEAR for Theme 4, and 3.1% in WPR to 6.3% in SEAR for Theme 5. AFR recorded the highest proportion of negative gains (37.7%), while the EMR had the lowest (7.4%). In terms of positive gains, the AMR predominantly reported Theme 2 gains (30.5%), whereas EUR had a higher proportion of Theme 3 gains (28.4%). AFR, EMR, SEAR, and WPR regions primarily observed Theme 1 gains, with proportions ranging from 38.0% to 45.6% (Figure S2 in the **Online Supplementary Document**).

#### Economic development levels

At different economic development levels, responses for Themes 1 to 5 varied: from 33.6% in UMICs to 44.9% in LICs for Theme 1, from 13.7% in LMICs to 25% in HICs for Theme 2, from 20.3% in HICs to 25.2% in LICs for Theme 3, from 10.1% in HICs to 14% in UMICs for Theme 4, and from 3.4% in LICs to 4.8% in UMICs for Theme 5. The LICs exhibited the highest proportion of negative gains (43.9%), while the LMICs reported the lowest (7.5%). Across all economic levels, Theme 1 was predominant, with proportions ranging from 33.6 to 44.9% (Figure S2 in the **Online Supplementary Document**).

## Discussion

In this qualitative study analysing 35 911 responses from 13 853 diverse international participants regarding their positive gains from the pandemic, we identified five positive gains from the COVID-19 pandemic (ranking from biggest to smallest response percentage): improved health awareness and practices, strengthened social bonds and trust, multi-dimensional personal growth, resilience and preparedness building, and accelerated digital transformation. The ranking reflects each theme's relative frequency and importance, enabling us to contextualise the qualitative findings by identifying the most and least prevalent gains. This allows policymakers and practitioners to prioritise high-impact initiatives and allocate resources effectively, while stakeholders can develop targeted strategies to reinforce strengths and address specific participant needs. These themes were consistently observed across demographics, with regional and cross-country variations: AMR focusing on social cohesion, EUR on personal development, and other regions on enhanced holistic health. Notably, 19 countries reported more gains in enhanced holistic health, five countries on strengthened social cohesion, and six on multi-dimensional personal development. A contrasting and unexpected theme of negative experiences, especially distrust and vulnerabilities, was more pronounced in countries like Burundi, Rwanda, and Canada, as well as African and low-income regions, significantly affecting non-binary individuals, those with lower education, and those facing employment barriers.

The pandemic has significantly increased health awareness and practices, surpassing previous expectations [19], and offers health care providers a chance to incorporate these into long-term plans. Quantitative analysis revealed that increased health knowledge, awareness, and practices were the most significant positive gains within this theme, requiring the highest attention. Public health campaigns should prioritise ongoing health awareness and hygiene practices to boost overall health and prepare for future crises by embedding pandemic-era habits – such as regular handwashing, mask-wearing during flu seasons, and improved sanitation protocols – into daily routines. This can be achieved through sustained educational programmes in schools and workplaces, reinforcing these practices within society. Additionally, health behaviour adaptations and lifestyle changes emerged as the second dominant subtheme. Reducing sugary drinks and increasing physical activity were key lifestyles [20,21], suggesting these areas should receive more support for holistic health improvements. Support measures might include taxing sugary beverages and developing public spaces that encourage physical activity, like parks and recreational centers. Moreover, health care systems are encouraged to advocate for a slower pace of life, emphasising rest and restoration, and promote work-life balance through policies such as paid mental health days, flexible working hours, and expanded telemedicine services. Increased environmental awareness presents an opportunity to promote eco-friendly habits and nature activities for better health. To translate environmental lessons into lasting behaviours, governments should invest in green urban spaces, support car-free zones, and implement community-based environmental programmes that encourage sustainable living. Public awareness campaigns should highlight the health benefits of reducing pollution and fostering a closer connection with nature, effectively transforming temporary pandemic gains into enduring societal changes that promote a healthier and more resilient society.

Strengthened social bonds and trust, beyond personal connections [11,22], offer health care systems an opportunity to develop lasting community resilience and health models, with strengthened relationships as the dominant subtheme. To leverage this, policies should support community-based health programmes, strengthen local networks, and create platforms for sharing experiences and health tips. The other two sub-theme are less dominant but still provide insights into an individual’s increased community responsibility and commitment as well as trust in authority, that can be leveraged for long-term benefits. Involving communities in health planning fosters active participation and civic engagement, which can be sustained by establishing community health committees or advisory boards that give residents a voice in health care decisions. Greater trust in health authorities can enhance guideline adherence and information sharing; thus, governments must maintain transparent, consistent communication strategies post-pandemic, including regular public health updates, open forums, and involving local leaders. This period of social solidarity provides a prime opportunity for collaborative health initiatives, like health ambassador programmes and mutual aid [23,24], encouraging shared health responsibility. To build on this, governments should institutionalise collaborative models, such as national health ambassador programmes, and encourage partnerships between businesses, civic organisations, and public health agencies to create a sustainable shared responsibility model.

Multi-dimensional personal growth, extending beyond previously reported self-organisation and hobbies [11,25,26], opens ways for adjustments in health and mental wellness initiatives. Quantitative analysis shows an even distribution among subthemes, suggesting balanced focus areas. Healthcare providers should design interventions that address both physical well-being and foster emotional intelligence and self-awareness. Programmes can include strategies for aligning personal values with health behaviours, such as mindfulness practices and value-driven goal-setting, promoting meaningful life changes. For instance, clinicians can help patients redefine their priorities in life, emphasising the importance of mental well-being and encouraging lifestyle adjustments that reflect personal values. Mental health services should adopt strategies that support emotional and philosophical realignment, aiding in self-understanding and resilience. Healthcare systems can utilise this shift towards empowerment and autonomy to foster patient-centred care, involving patients in their health decisions. Health programmes addressing tangible personal growth, like time management and skill acquisition, can promote a balanced lifestyle, integrating professional and personal well-being. Educational efforts in health care could emphasise skills and hobbies, such as yoga, for overall well-being and life satisfaction.

Amidst the pandemic's extensive losses [27], society has cultivated widespread resilience and preparedness building. Acceptance, coping, and resilience, along with financial dynamics and management, were the two dominant subthemes. Healthcare systems can harness this by offering programmes focused on coping strategies and positive psychology, thereby sustaining and enhancing these developments and preparing communities for future challenges. Health education should emphasise crisis management, emergency preparedness, and life strategy planning to seize their forward-thinking mindset. To ensure lasting preparedness, public health campaigns should teach practical crisis management and proactive planning skills, including financial literacy, disaster preparedness, and mental resilience through schools, community workshops, and workplace training. Governmental aid in crises can further reinforce societal resilience by establishing robust support systems and emergency funds. These initiatives can solidify the behavioural advancements into long-term societal resilience.

Expanding on previous localised or group-specific digital transformation research [28–30], our study further validates these benefits. Online learning is the primary aspect of digital transformation, necessitating blending digital health education into curricula or training to ensure digital equity. Educational systems should teach digital skills early, emphasising not only basic technology use but also critical thinking, digital safety, and online communication. Remote work is the second major facet, requiring enhancements in telehealth and addressing remote workers' health issues, such as ergonomics and eye strain. Policymakers should implement workplace wellness guidelines targeting these risks, including ergonomic training and mental health resources. Employers can adopt hybrid models with flexible arrangements and provide resources for mental and physical well-being, such as virtual ergonomic consultations or mindfulness workshops [31]. Other subthemes vary in the extent of transformation. Growing digital literacy and increased digital communication are paving the way for advanced digital health tools, including mental health platforms, telemedicine services, and mobile health apps. Governments and health organisations should invest in creating user-friendly, evidence-based digital health tools for widespread adoption. For example, developing accessible mental health apps with personalised support can expand care access, especially in remote or underserved areas. The rise in e-commerce suggests digital channels for health products and services. Health systems can partner with e-commerce platforms to deliver medical supplies, over-the-counter medications, and wellness products directly to consumers. Additionally, health care providers should implement telemedicine for routine check-ups, prescription refills, and consultations, reducing the need for in-person visits. Overall, this digital transformation highlights the necessity for health systems to adapt by providing flexible, technology-enhanced services and policies aligned with the digital age. Governments should support digital infrastructure development, particularly in rural or underserved regions, to ensure universal access to these services.

While our study primarily focused on positive outcomes from the COVID-19 pandemic, some participants reported negative gains, which we retained for their valuable insights. The proportion of negative gains indicates the extent to which regions or groups benefited from the pandemic. Areas with high negative responses, like Burundi (94.1%), may have missed opportunities for positive advancements, suggesting a need to prioritise recovery strategies over positive transformation. In regions dominated by negative gains, the focus should shift to addressing vulnerabilities and facilitating recovery. Beyond previous findings [1,32–34], our findings highlight the emergence of new vulnerabilities and a decline in public trust as key negative themes. Emotional challenges were the most dominant subtheme, followed by unmet basic needs and distrust in authorities. Given the compounded physical and emotional hardships, continuous care is essential. To tackle these issues, health care systems should implement community-based health and wellness programmes focused on mental health recovery and providing ongoing physical care, especially in trauma-affected regions. Rebuilding public trust requires transparent health care decision-making and effective communication strategies, including involving trusted community leaders and creating participatory platforms for public engagement. Additionally, providing affordable health care options, including regionally accepted traditional medicine, can alleviate financial burdens. Addressing unmet basic needs such as food insecurity, poverty, theft, and crime necessitates community-centred programmes that offer financial support, job training, and access to social services.

Quantitative analysis reveals thematic variations across regions, countries, and demographics, necessitating customised health strategies. In low- and middle-income areas such as AFR, EMR, SEAR, WPR, and 19 countries including Indonesia with underdeveloped health infrastructures [35]. The COVID-19 pandemic heightened health practices, leading to substantial improvements in health awareness and practices, resulting in the highest response percentage in the enhanced holistic health theme. In these contexts, health policies that specifically target holistic health could maximise health outcomes. In the AMR region and countries such as Chile, where individualism is prioritised [36], increased family time during pandemic-induced social distancing led to significant responses in the enhanced social cohesion theme, suggesting that community-focused health initiatives would be particularly effective. Similarly, in EUR and nations like Spain, the emphasis on personal development-backed by rich history, linguistic diversity, and cultural traditions in arts and philosophy-facilitates positive health transformations. This results in high responses for multi-dimensional personal growth, indicating that policies promoting individual health development could be particularly effective. Thailand's achievements in digital and resilience areas highlight the value of enhancing these strengths [37], whereas Burundi's digital gaps-evidenced by zero responses in this theme-are due to limited internet access, low digital literacy, economic constraints, and political and regulatory challenges [38,39]. Addressing these gaps requires investing in internet infrastructure, implementing digital literacy programmes, providing economic support for digital investments, and developing supportive policies and regulations.

### Limitations and future research

Several limitations should be noted. First, while convenience sampling allowed us to collect data from a diverse range of participants across 30 countries, it has led to overrepresentation of certain demographic groups and regions, especially the young and female participants, as well as a limited number of respondents from low-income countries. This demographic skew may have biased the thematic distribution, potentially limiting the generalisability of our findings and highlighting the need for more balanced sampling methods in future studies. Second, we have not yet formulated a strategy to leverage the positive outcomes towards building a more sustainable and resilient society. Future research should explore methods to capitalise on these benefits for societal betterment. It should aim at identifying specific strategies and interventions that can transform these gains into actionable policies and support for communities. Third, this study was specifically designed to focus on positive gains from the COVID-19 pandemic. Although negative gains emerged unexpectedly and were retained due to their valuable insights, the survey was not structured to systematically capture negative outcomes. Consequently, the ratio between positive and negative gains does not reflect their true prevalence or significance and should not be interpreted as indicative of their relative importance. Fourth, while this study integrates quantitative methods to enable straightforward comparisons between regions and demographic groups, facilitating more accessible and actionable policy guidance, using ‘percentage of responses’ in thematic analysis may oversimplify qualitative data and introduce potential biases, particularly in regions with small sample sizes. Fifth, relying on a single open-ended question may have limited the depth of responses and overlooked key aspects of participants’ experiences. Future research should use more comprehensive qualitative methods, such as interviews or focus groups. Sixth, this study does not account for the complex cultural and political factors that may influence thematic variations, as these elements are multi-dimensional and data availability is limited, thereby constraining the depth of our comparative analysis.

## CONCLUSIONS

In summary, this study reveals the COVID-19 pandemic's varied positive gains, including enhanced holistic health, strengthened social cohesion, multidimensional personal growth, increased resilience and adaptation, and significant digital shifts. These findings offer a balanced view of the pandemic's impact and provide insights to inform future health policies aimed at making these pandemic-induced positive shifts more sustainable, healthier, and resilient. Additionally, the emergence of an unexpected 'negative gains' theme, which highlights social instability, new vulnerabilities, and distrust, warrants targeted interventions for recovery. Notably, the variation in thematic responses across demographics, countries, and regions reflects both the ability to harness positive changes and the severity of negative impacts, necessitating tailored health strategies. Future research should focus on longitudinal studies to assess the sustainability of these positive changes over time and comparative studies across diverse cultural contexts to understand how cultural, economic, and health care system factors influence these gains. Additionally, employing mixed-methods approaches and targeting underrepresented populations will ensure a more comprehensive and inclusive understanding of the pandemic's impact.

## Additional material


Online Supplementary Document
