# Supplementary material for: Individuals’ positive gains from the COVID-19 pandemic: a qualitative study across 30 countries
Source: J Glob Health. 2025 Feb 14;15:04091. doi: 10.7189/jogh.15.04091 (PMC11827041; doi:10.7189/jogh.15.04091)

ONLINE SUPPLEMENTARY DOCUMENT

Title: Individuals' Positive Gains from the COVID-19 Pandemic: A Qualitative Study Across 30 Countries

Authors: Jiaying Li, Patricia M Davidson, Yaqin Li, Daniel Yee Tak Fong, Kris Yuet Wan Lok, Janet Yuen Ha Wong, Mandy Man Ho, Edmond Pui Hang Choi, Vinciya Pandian, Wenjie Duan, Marie Tarrant, Jung Jae Lee, Chia-Chin Lin, Oluwadamilare Akingbade, Khalid M Alabdulwahhab, Mohammad Shakil Ahmad, Mohamed Alboraie, Meshari A Alzahrani, Anil S Bilimale, Sawitree Boonpatcharanon, Samuel Byiringiro, Muhammad Kamil Che Hasan, Luisa Clausi Schettini, Walter Corzo, Josephine M. De Leon, Anjanette S. De Leon, Hiba Deek, Fabio Efficace, Mayssah A El Nayal, Fathiya El-Raey, Eduardo Ensaldo-Carrasco, Pilar Escotorin, Oluwadamilola Agnes Fadodun, Israel Opeyemi Fawole, Yong-Shian Shawn Goh, Devi Irawan, Naimah Ebrahim Khan, Binu Koirala, Ashish Krishna, Cannas Kwok, Tung Thanh Le, Daniela Giambruno Leal, Miguel Ángel Lezana-Fernández, Emery Manirambona, Leandro Cruz Mantoani, Fernando Meneses-González, Iman Elmahdi Mohamed, Madeleine Mukeshimana, Chinh Thi Minh Nguyen, Huong Thi Thanh Nguyen, Khanh Thi Nguyen, Son Truong Nguyen, Mohd Said Nurumal, Aimable Nzabonimana, Nagla Abdelrahim Mohamed Ahmed Omer, Oluwabunmi Ogungbe, Angela Chiu Yin Poon, Areli Reséndiz-Rodriguez, Busayasachee Puang-Ngern, Ceryl G Sagun, Riyaz Ahmed Shaik, Nikhil Gauri Shankar, Kathrin Sommer, Edgardo Toro, Hanh Thi Hong Tran, Elvira L Urgel, Emmanuel Uwiringiyimana, Tita Vanichbuncha, Naglaa Youssef

Supplementary materials

eFigures	Page number
**eFigure 1.** Proportional distribution of negative to all responses and positive by theme to all positive, by demographics (n = 35,911).	1
**eFigure 2.** Proportional distribution of negative to all responses and positive by theme to all positive, by WHO region and economic development level (n = 35,911).	2

eFigure 1. Proportional distribution of negative to all responses and positive by theme to all positive, by demographics (n = 35,911).

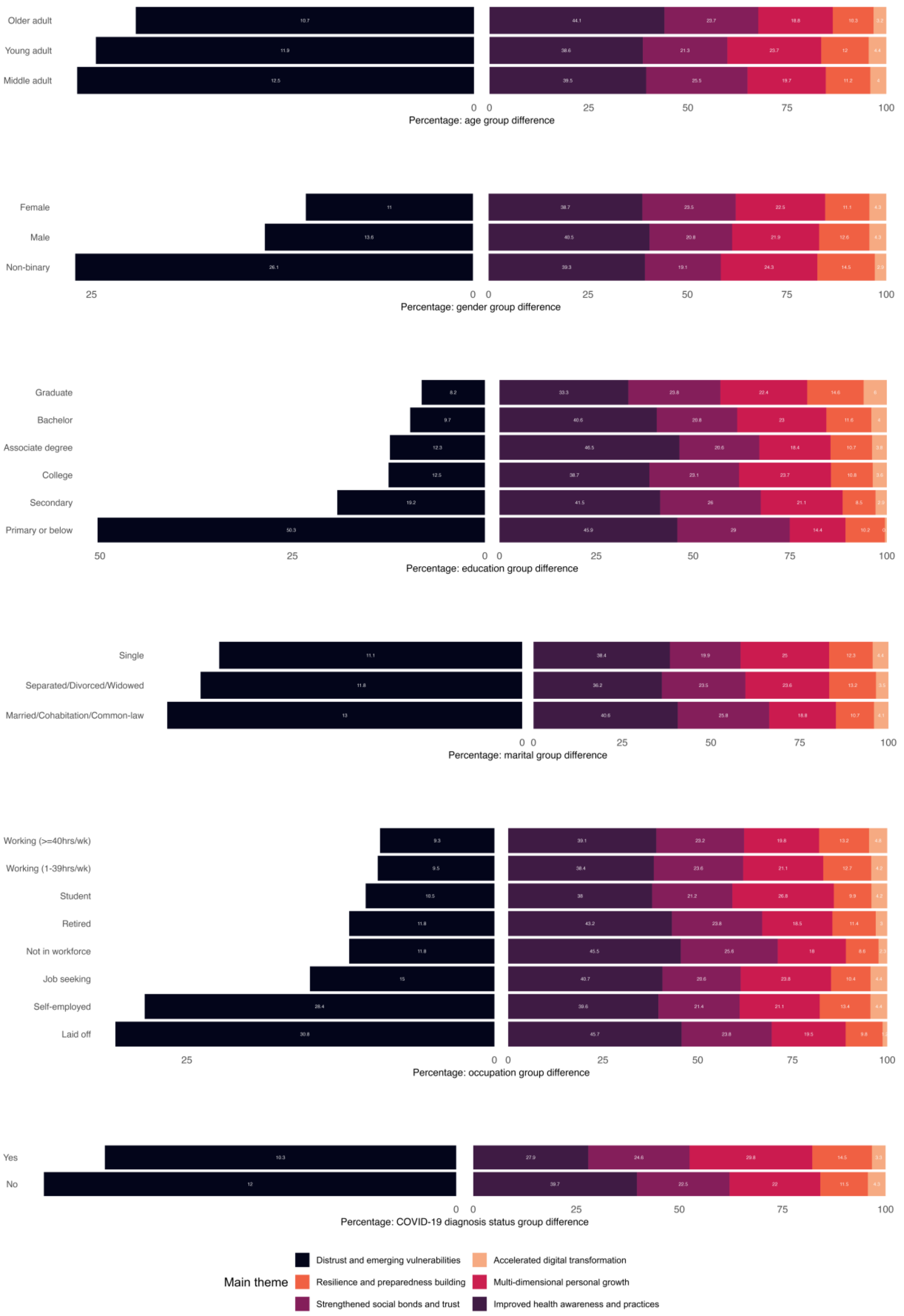

eFigure 2. Proportional distribution of negative to all responses and positive by theme to all positive, by WHO region and economic development level (n = 35,911).

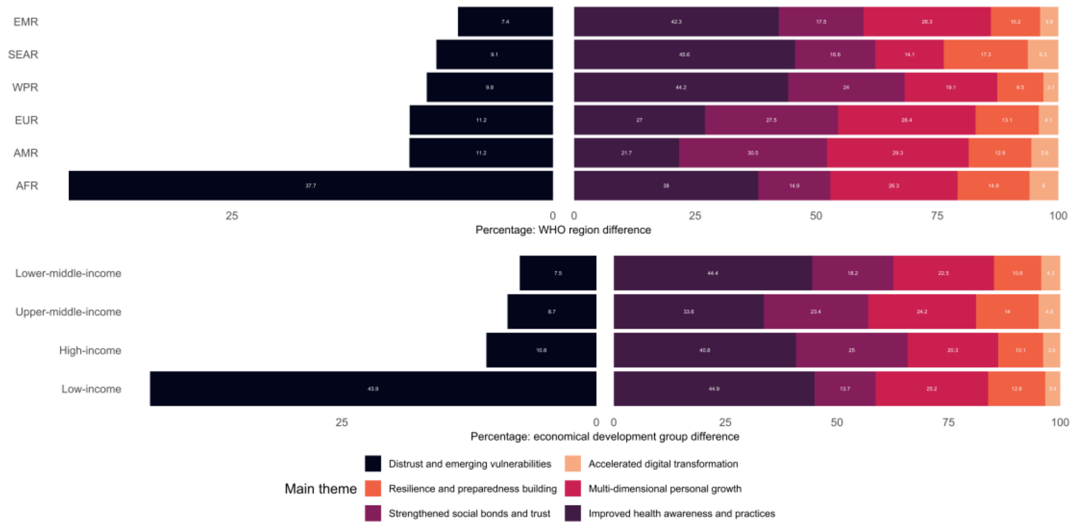

Supplement: Online Supplementary Document [file jogh-15-04091-s001.pdf]
